# MUSIC: identification of enriched regions in ChIP-Seq experiments using a mappability-corrected multiscale signal processing framework

**DOI:** 10.1186/s13059-014-0474-3

**Published:** 2014-10-08

**Authors:** Arif Harmanci, Joel Rozowsky, Mark Gerstein

**Affiliations:** Program in Computational Biology and Bioinformatics, Yale University, 260 Whitney Avenue, New Haven, CT 06520 USA; Department of Molecular Biophysics and Biochemistry, Yale University, 260 Whitney Avenue, New Haven, CT 06520 USA; Department of Computer Science, Yale University, 260 Whitney Avenue, New Haven, CT 06520 USA

## Abstract

We present MUSIC, a signal processing approach for identification of enriched regions in ChIP-Seq data, available at music.gersteinlab.org. MUSIC first filters the ChIP-Seq read-depth signal for systematic noise from non-uniform mappability, which fragments enriched regions. Then it performs a multiscale decomposition, using median filtering, identifying enriched regions at multiple length scales. This is useful given the wide range of scales probed in ChIP-Seq assays. MUSIC performs favorably in terms of accuracy and reproducibility compared with other methods. In particular, analysis of RNA polymerase II data reveals a clear distinction between the stalled and elongating forms of the polymerase.

**Electronic supplementary material:**

The online version of this article (doi:10.1186/s13059-014-0474-3) contains supplementary material, which is available to authorized users.

## Background

With the recent advancements in sequencing technologies, chromatin immunoprecipitation (ChIP)-based enrichment of DNA sequences followed by sequencing (ChIP-Seq) [1,2] has become the mainstream experimental method for genome-wide measurement of the locations of DNA binding proteins like transcription factors (TFs) and posttranslational modifications of histone proteins, or histone modifications (HMs) [3,4]. Consortium projects such as ENCODE [5] and the Roadmap Epigenomics Project [6] generated ChIP-Seq datasets to map the chromatin states of many cell lines and tissues [7]. These substantially increased the number of publicly available ChIP-Seq datasets for a diverse set of HM and TF binding profiles. Following sequencing, it is necessary to computationally process the read depth (RD) signal profile to identify the enriched regions (ERs) across the genome [8].

Depending on the target of the ChIP-Seq assay, the length scale of ERs can vary extensively for different experiments, which changes the ER identification workflow. For TF binding, for example, the ERs are observed at punctate regions of protein binding and are hundreds of nucleotides in length [9]. For most HMs, ERs are broad. For example, the ERs for the repressive heterochromatin mark H3K9me3 can extend up to a few megabases. Another interesting example is RNA polymerase II (Pol2), which binds to promoters and gene bodies for the purpose of mRNA transcription and whose ERs can extend over the whole gene bodies or can be punctate and concentrated close to gene promoters. Development of efficient computational methods for identification and characterization of the broad ERs is necessary for understanding the regulatory effects of HMs and diffuse DNA binding proteins on gene expression as increasing evidence indicates that these epigenetic factors are major driving factors in pluripotency [10] and of disease manifestation, such as cancerogenesis [11-15].

There are two main challenges for identification of broad ERs. First, unlike ERs for TF binding, broad ERs are observed at longer length scales and the length spectrum of ERs is broad for many HMs. This makes it necessary to identify the ERs at different scales. A widely used method for identifying ERs in HM signal profiles involves smoothing the signal profile with a kernel of constant size and shape and using a null model (for example, a Poisson or negative binomial) to identify the significantly enriched regions. It is, however, not clear how the kernel size and shape should be selected. The multiscale approaches proposed by the wavelet-based methods address this issue, although the reasoning and motivation for which wavelet functions are used in these methods are generally not well established.

Second, the signal profiles contain systematic noise introduced into the RD signal by repeat regions with low mappability [9,16], in the form of loss of signal. This noise causes discontinuities in the identified ERs. This is an important factor, especially in intergenic regions where a large ER, which may mark a long regulatory region, is fragmented into smaller ERs.

Many different approaches have been applied for identification of broad ERs, including change point identification within the formality of Bayesian inference (BCP [17]), local island identification and clustering (SICER [18]), local thresholding and merging (MACS), using local Poisson statistics to identify broad ERs (SPP), and wavelet-based smoothing and identification of ERs (WaveSeq [19]), which is also applied to analysis of ChIP-chip datasets [20].

In this paper, we present MUSIC, a method to identify ERs in ChIP-Seq experiments. MUSIC first uses mappability correction at nucleotide resolution to correct for the spurious loss of signal in regions with low mappability. Next, MUSIC performs a multiscale decomposition of the corrected RD signal. This decomposition is adopted from the scale-space filtering theory in signal processing [21], which is used widely for signal segmentation, smoothing, and enhancement. Unlike the wavelet-based multiscale approaches that use linear filtering, we take an approach to multiscale decomposition that uses non-linear median filtering. Basically, MUSIC exploits the fact that, at each decomposition, smoothing with a certain window length removes small details in the signal (like small peaks and small valleys) and the candidate ERs in the signal are detected as regions between consecutive local minima of the smoothed signal [22,23]. MUSIC then identifies the significantly enriched regions at each scale, which yields the scale-specific ERs (SSERs). In general, SSERs at smaller scales correspond to more punctate binding/modification levels compared with SSERs at higher scales, which represent broader ERs. To identify the final set of ERs, MUSIC merges the SSERs from all the scales.

In order to evaluate the accuracy of the ERs identified by MUSIC, we performed benchmarking experiments to compare the accuracy and reproducibility of the ERs identified by MUSIC with numerous other ER identification methods. We concentrated on factors whose ERs manifest at different (that is, broad, puncate, and point binding) length scales so as to make a thorough comparison with a variety of accuracy metrics. We show that MUSIC performs favorably in the comparisons. Next, we concentrate on the Pol2 ChIP-Seq datasets. Motivated by the basic observation that the stalled polymerase tends to show punctate enrichments (SSERs at small scales) and that elongating polymerase tends to show broad enrichments (SSERs at higher scales), we computed the SSERs for the Pol2 ChIP-Seq dataset using MUSIC. Using the identified SSERs, we then estimate the length scale for polymerase binding for all protein coding genes. We demonstrate that the genes with punctate polymerase binding have significantly lower expression (close to 0) than the genes that show more broadly bound polymerase. We corroborate this observation with the ChIP-Seq data for the elongating (phosphorylated) form of Pol2. We conclude that the length scale of binding of polymerase at gene promoters as identified by MUSIC is indicative of its state, that is, stalled or elongating.

The paper is organized as follows. We first present the MUSIC algorithm and lay out the steps of the algorithm. Then we present a comparison of MUSIC with other ER identification algorithms. We finally present the analysis of the Pol2 data with gene expression levels.

## Results and discussion

### MUSIC algorithm

**Figure 1:**
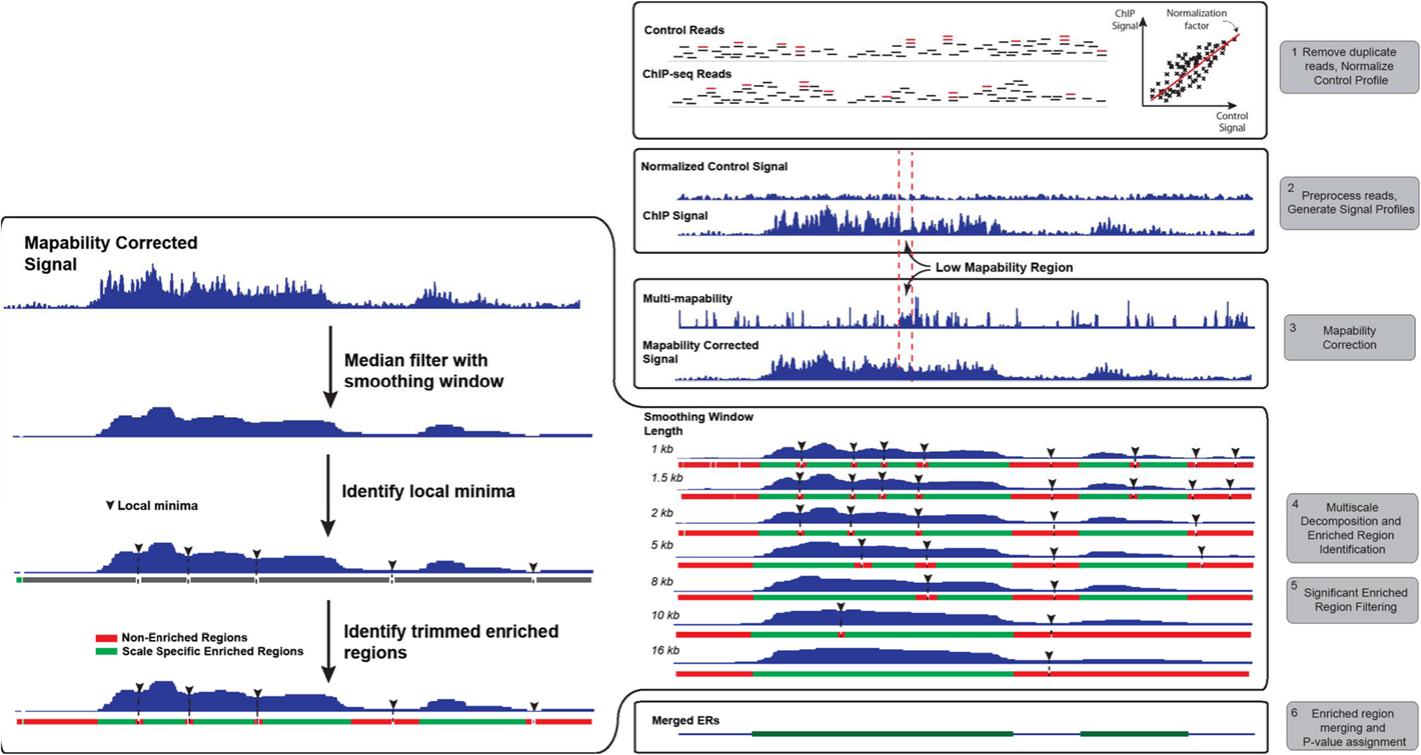
**Flowchart of MUSIC.** H3K36me3 ChIP and control data in the region chr1:55,170,679-55,240,996 of the K562 cell line is used for illustrating the signal processing steps. ChIP and control reads (represented by short horizontal lines) are filtered for duplicates (red colored) and control signal is normalized with respect to ChIP signal (step 1). RD profiles are generated (step 2). The ChIP-Seq profile is corrected for mappability (labeled 'Mappability Corrected Signal') using the multi-mappability profile. Note the region indicated between the dashed lines that has low signal because of low mappability is filled with correction (step 3). Seven scale decomposition of the ChIP-Seq signal is computed. Under each decomposition, the ERs with the corresponding local minima are shown (step 4). The connected window shows the processing performed for generating the SSERs at each scale. The mappability-corrected signal is smoothed, the local minima are identified and the candidate ERs are formed (shown in grey), then candidate ERs are trimmed and filtered (shown in red) with respect to significance to identify the SSERS (shown in green.) SSERs for each scale are shown under the corresponding decomposition (step 5). The final set of ERs is formed by merging the SSERs (step 6).

Figure 1 shows a flowchart for MUSIC (see Materials and methods for more details). Here we summarize each step briefly. The input to MUSIC comprises the sets of reads from the ChIP and control samples (steps 1 and 2), the set of window lengths to be used in multiscale decomposition, and the multi-mappability profile. The multi-mappability profile quantifies, at each position, the average number of reads that get mapped non-uniquely (see Materials and methods). Therefore, for a position that is uniquely mappable, the multi-mappability value is 1. For repeat regions, the multi-mappability value increases. Figure S1 in Additional file 1 shows the aggregation of multi-mappability profiles around different genomic elements for different read lengths. It should be noted that the multi-mappability signal is computed once for each read length (see the 'Multi-mappability signal generation' section in Materials and methods). MUSIC first preprocesses the reads and filters the duplicates, then computes a scaling factor using linear regression between the ChIP and control signal profiles (see the 'Input normalization' section in Materials and methods). The slope of the regression is used as a normalization factor for control.

Then, in step 3 in Figure 1, the ChIP and normalized control signal profiles are generated, and the ChIP profile is filtered and corrected with respect to mappability using the multi-mappability profile (see the 'Mappability correction filter' section in Materials and methods). The correction can be formulated as:

$$ {\tilde{x}}_i=\overset{\begin{array}{c}\mathrm{Maximum}\ \mathrm{of}\ \mathrm{the}\ \mathrm{s}\mathrm{ignal}\ \mathrm{value}\ \mathrm{at}\ i\ \mathrm{and}\\ {}\mathrm{the}\ \mathrm{median}\ \mathrm{s}\mathrm{ignal}\ \mathrm{at}\ \mathrm{highly}\ \mathrm{mappable}\ \mathrm{positions}\end{array}}{\overbrace{ \max \left[{x}_i,\underset{\begin{array}{c}\mathrm{Median}\ \mathrm{of}\ \mathrm{the}\ \mathrm{s}\mathrm{ignal}\ \mathrm{value}\mathrm{s}\ \mathrm{at}\ \mathrm{highly}\ \mathrm{mappable}\ \\ {}\mathrm{positions}\ \mathrm{around}\ i\end{array}}{\underbrace{\mathrm{median}\left({\left\{{x}_a\right\}}_{a\in \left[i-{l}_c/2,\ i+{l}_c/2\right]}\ \Big|\ {m}_a<{\overline{m}}_{\mathrm{exonic}}\right)}}\right]}} $$

where *x*_*i*_ and ${\tilde{x}}_i$ are the uncorrected and corrected signal values, respectively, at position *i*, *m*_*a*_ is the value of multi-mappability profile at position *a*, *l*_*c*_ is the length of the median filter utilized in correction, which is by default set to 2,000 bp, and ${\overline{m}}_{\mathrm{exonic}}$ is the average multi-mappability signal value over the exonic regions, which we identified as the most mappable regions in the genome (Figure S1 in Additional file 1). In summary, for each position *i*, MUSIC computes the median of the signal values at highly mappable positions (multi-mappability signal smaller than ${\overline{m}}_{\mathrm{exonic}}$) within *l*_*c*_ vicinity of *i*. Then MUSIC compares this value with the signal value at *i* and assigns the maximum to the corrected value. The basic idea behind this correction is that since we know that low mappability causes a decrease in the signal level, if the signal value at *i* is higher than its vicinity, then it is highly likely that the mappability did not affect the signal value at *i*. Otherwise, it is replaced by the median signal value at mappable positions. The maximum filtering, also known as dilation in the image processing literature, is used for feature enhancement in images [24], which also enables MUSIC to enhance the ERs for easier identification. It should be noted that the mappability correction is not required for correcting the signal profiles of very punctate signal profiles like TFs since TF peaks in the signal profile have predominantly single summits, which do not get segmented by regions with low mappability.

MUSIC then performs median filtering to the mappability-corrected ChIP profile to compute multiscale decomposition of the ChIP signal at multiple length scales (step 4 in Figure 1; Figure S2 in Additional file 1; 'Multiscale decomposition by median filtering' section in Materials and methods). For this, MUSIC uses window lengths beginning with *l*_*start*_ and ending at *l*_*end*_ and performs sliding window-based median filtering. The window length is increased multiplicatively between consecutive scales; thus, the window lengths form a geometric series:

$$ \left\{{l}_{start}\left\lfloor,, {l}_{start}\times \sigma \right\rfloor, \left\lfloor {l}_{start}\times {\sigma}^2\right\rfloor, \cdots, {l}_{end}\right\} $$

where *σ* is the multiplicative factor between consecutive window lengths, which is set to 1.5 by default. ⌊*l*_*start*_ × *σ*⌋ denotes the largest integer value that is smaller than *l*_*start*_ × *σ*, which is necessary since the window lengths are integer values. The multiplicative factor tunes how finely MUSIC samples the scale spectrum. For small *σ*, MUSIC analyzes large numbers of scale lengths, although this also increases the run time.

For smoothed signal at each scale, MUSIC identifies all the local extrema, that is, local minima and local maxima (step 4 in Figure 1; 'Identification of candidate scale-specific enriched regions' section in Materials and methods). The regions between the consecutive local minima are marked as the candidate ERs. Due to the nature of the smoothing process, the signal may become oversmoothed at large scales (long windows), which causes over-merging of the ERs. To avoid this, it is necessary to remove the regions with over-smoothed signal. For each ER, MUSIC computes the fraction of the maximum of smoothed RD signal (at the corresponding scale) to the maximum of the unsmoothed ChIP signal within the boundaries of the ER. If this fraction is smaller than the smoothed versus unsmoothed signal ratio threshold (denoted by *γ*), MUSIC discards this candidate ER (see the 'Comparison of smoothed signal in candidate enriched regions' section in Materials and methods).

The regions identified from the consecutive minima are rough and it is necessary to identify the location of the densest signal enrichment within each region. To achieve this, MUSIC performs a Poisson background-based thresholding and *P*-value minimization to trim the ends and identify the densest regions of signal enrichment in the ERs. Step 5 in Figure 1 illustrates the trimmed ends of the candidate ERs. Finally, MUSIC computes the *P*-value from a binomial test for each trimmed region and filters out those whose *P*-values are larger than 0.05. We refer to the remaining regions as scale-specific enriched regions; these contain all the information about the enrichments in the signal over a spectrum of length scales (see the 'Candidate enriched region end trimming using a Poisson distribution model' and 'Candidate enriched region end trimming via *P*-value minimization' sections in Materials and methods).

#### Identification of enriched regions

MUSIC utilizes SSERs to identify ERs in the genome. For this, the candidate ERs are computed by merging the SSERs identified in all the scales (step 6 in Figure 1). MUSIC then filters out the ERs with respect to discordance of the signal levels on positive and negative strands. For this, MUSIC computes the amount of signal mapping to the positive and negative strands in each ER and filters out the ERs for which the counts of reads that map to the positive and negative strands are within a factor of 2 of each other (see the 'Per strand concordance test' section in Materials and methods).

For each of the remaining ERs, MUSIC computes the *P*-value from a binomial test using the number of reads in the ChIP and normalized control samples. The multiple hypothesis correction is performed by the Benjamini-Hochberg procedure [25]. The q-values computed after the correction are thresholded with respect to 0.05 for identification of the significant ERs (see the '*P*-value computation and false discovery rate estimation' section in Materials and methods). For each ER, MUSIC also computes a summit (see the 'Summit and trough identification' section in Materials and methods) and a trough in the ER. The summits represent the point of strongest binding/modification in the ER and troughs represent the point where there is a depletion of signal, which may represent the nucleosome-free regions. Finally, in order to visualize the processed tracks, MUSIC has an option to save the smoothed signal profiles at each decomposition scale in bedGraph format, which can be loaded to a genome browser.

#### SSER pileup scale and evaluation of broadness of enrichment

The scale dependence of SSERs is a useful property for evaluating the broadness of enrichment. Each SSER represents a local ER at a certain length scale. Therefore, the signal around a position that is covered by a large number of SSERs (at different scales) is more broadly enriched than the signal around a position that is covered by fewer SSERs. Following this basic observation, MUSIC pools the SSERs from all the scales and counts the number of SSERs covering each position, which quantifies the broadness of enrichment at each position in the genome. We refer to this value as the SSER pileup scale of the position.

**Figure 2:**
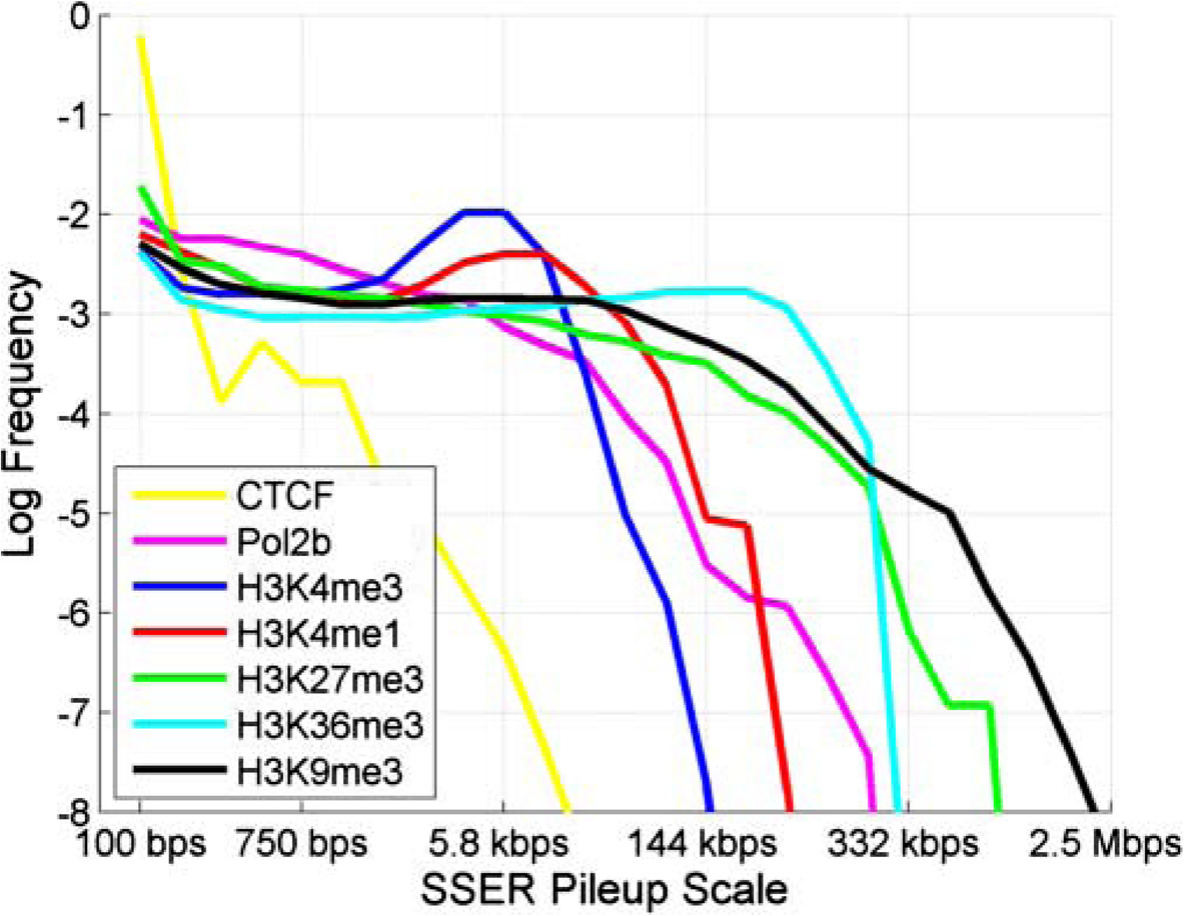
**Distribution of the pileup scale.** Distribution of SSER pileup scale for CTCF, Pol2b, and several different HMs. The length scale is between 100 bp and 2.5 Mbp as shown on the x-axis. The y-axis shows the log frequency.

To evaluate the spectrum of enrichment length scales specific to different datasets, we processed multiple ChIP-Seq datasets (CTCF, Pol2, H3K4me1, H3K4me3, H3K36me3, H3K27me3, and H3K9me3) from the ENCODE project for the K562 cell line with window length parameters *l*_*start*_ = 100 bp, *l*_*end*_ = 2.5 Mbp, and *σ* = 1.5 (total of 25 scales) and computed the SSER pileup scales for the positions on chromosome 1. Figure 2 shows the distribution of SSER pileup scales, that is, the pileup scale spectrum of all the positions on chromosome 1 for different datasets. We use this plot to assess the scale length characteristics of different datasets. CTCF, a punctate binding TF, has a maximum frequency at the smallest pileup scales compared with the other datasets. This suggests, as expected, that CTCF has the most punctate ERs compared with the other datasets. H3K4me3 and H3K4me1, active promoter and enhancer HM marks, show broader enrichments than CTCF. H3K36me3 and H3K27me3, which mark active and repressed gene bodies, show broader enrichments, and H3K9me3, an HM-associated with large heterochromatin domains, shows the broadest enrichments. Another interesting observation is that the plots for the H3K4me3, H3K4me1, and H3K36me3 datasets have maxima at certain scales, which indicates that these HMs are enriched at specific length scales that are observed frequently. Figure S7 in Additional file 1 shows the scale spectrum for a more extensive list of HMs with corresponding length scales. Finally, the Pol2 signal profiles show a high frequency of enrichments at small scales that gradually decreases as the scale increases.

### Comparison with other methods

In order to evaluate the accuracy of the ERs, we compared MUSIC with eight other algorithms that identify ERs from ChIP-Seq data: DFilter [26], ZINBA [27], F-Seq [28], BCP [17], SPP [29], MACS [30], SICER [18], and PeakRanger [31]. A detailed list of the parameters used to run each method are presented in the 'Parameter selection for benchmarking' and 'Parameters used for peak calling methods in benchmarking' sections in Materials and methods.

#### Comparison of broad enriched region identification

To compare the performance of the methods on broad marks, we ran all the algorithms (in broad ER identification mode) using H3K36me3 and H3K27me3 ChIP-Seq datasets for GM12878 and K562 cell lines from the ENCODE project [5]. H3K36me3 is known to mark the bodies of actively transcribed genes [32]. We used this observation to build a gold standard set for H3K36me3 comprising the bodies of expressed transcripts. We downloaded the transcript quantifications (in Reads Per Kilobase per Million mapped reads, RPKM ) from Djebali *et al*. [33] and removed transcripts with low expression. The bodies of the expressed transcripts were then merged to generate the gold standard set for H3K36me3 ERs. Rather than selecting one expression threshold for identifying the expressed transcripts, we selected thresholds between 0 and 1 RPKM increasing in steps of 0.01 so as to evaluate the accuracy of ER calls against multiple gold standard sets identified at different levels of gene expression.

We observed that MUSIC tends to identify longer ERs compared with other methods and that different methods have very different total ER coverage. To measure the accuracy of identified ERs, it is necessary to account for the difference in the coverage of the identified ERs. We used sensitivity (the fraction of the coverage of correctly predicted ERs to the coverage of the gold standard set) and positive predictive value (the fraction of the coverage of correctly predicted ERs to the coverage of identified ERs). To summarize these accuracy values in one measure, we chose the F-measure, which is computed as the harmonic mean of sensitivity and positive predictive value (see the 'Parameter selection for a new ChIP-Seq dataset' section in Materials and methods). Having one measure of accuracy enables us to easily compare the accuracy of methods with changing RPKM thresholds.

**Figure 3:**
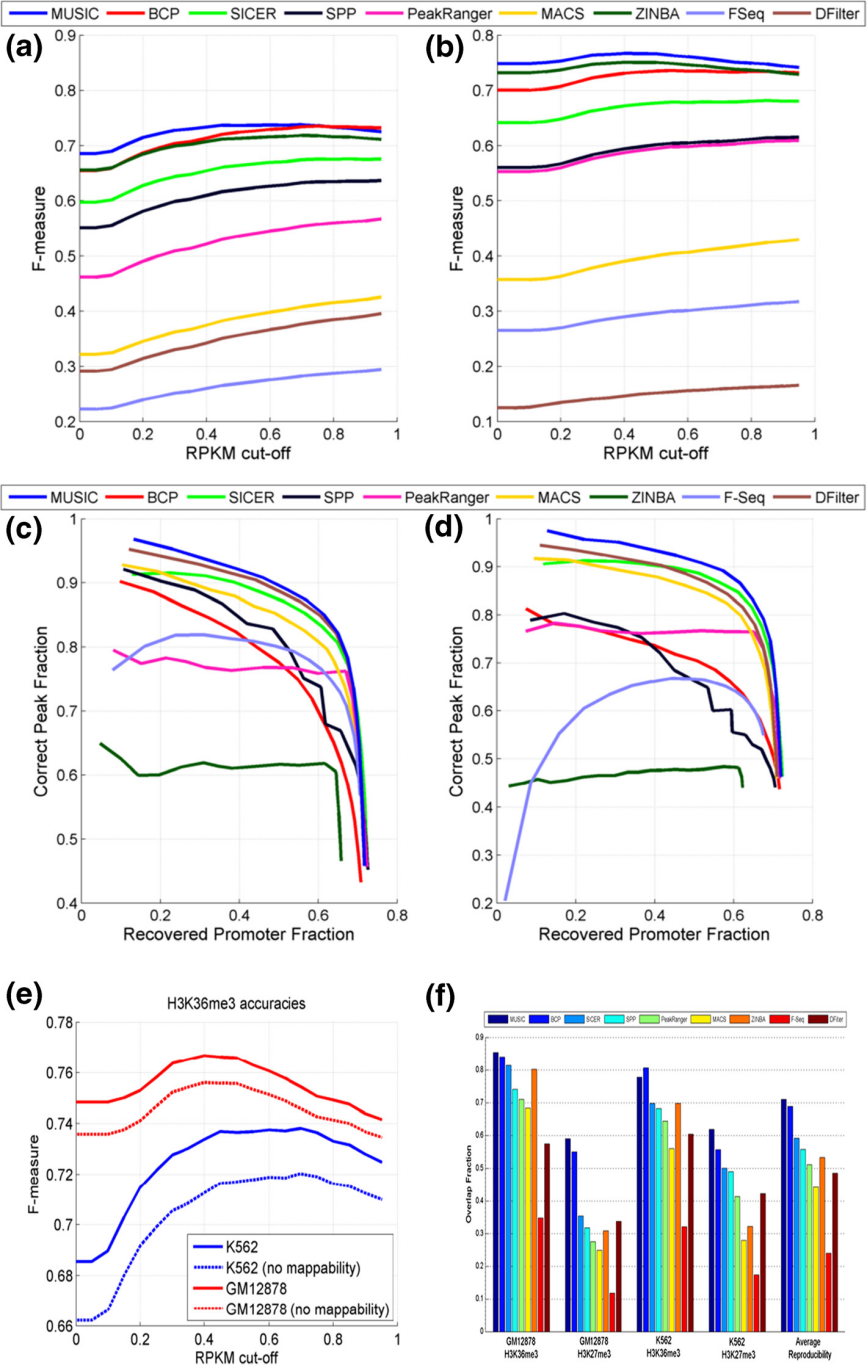
**Accuracy comparison for predicted H3K36me3 ERs. (a,b)** F-measure versus RPKM threshold for H3K36me3 ERs for GM12878 **(a)** and K562 **(b)** cell lines. **(c,d)** The recovered promoter fraction versus correct peak fraction for H3K4me3 ERs for GM12878 **(c)** and K562 **(d)** cell lines with increasing peak rank. **(e)** The F-measure versus RPKM cutoff with (red) and without (blue) mappability correction. **(f)** The reproducibility of the H3K36me3 and H3K27me3 ERs. The average overlap fraction is shown on the y-axis. Average reproducibility for each method is computed as the average over all the fractions.

Figure 3a,b shows the F-measure for H3K36me3 ERs from the different methods with respect to the changing RPKM cutoffs. MUSIC resulted in a higher F-measure than all the other methods for GM12878 at all expression cutoffs, followed by BCP. For K562, MUSIC resulted in a higher F-measure than all other methods for expression cutoffs <0.8 RPKM, falling slightly below BCP for expression cutoffs >0.8 RPKM. For assessing the importance of mappability correction, we ran ER identification without mappability correction and computed the F-measure of the ERs. Figure 3e shows the F-measure versus RPKM threshold. Using a mappability map significantly increases the accuracy of the identified ERs for H3K36me3 and shows the importance of utilizing the mappability correction in ER identification.

We also evaluated the reproducibility of the ERs. For this comparison, we used the replicates generated by the ENCODE project. For H3K36me3 and H3K27me3, we computed the reproducibility as the average of the fraction of the overlapping regions to the total coverage of each replicate (see Materials and methods; Figure 3f). The overall reproducibility for MUSIC is higher than that for the other methods and MUSIC has the best or the second best reproducibility compared with the other methods.

#### Comparison of punctate enriched region identification

While the multiscale approach developed in MUSIC is most applicable to identifying ERs over a range of length scales, it can be applied to the identification of punctate ERs, such as for TFs. To compare the methods listed above in the identification of punctate ERs, we first chose to compare the methods on the H3K4me3 HM, which marks the promoters of active genes. We utilized the promoters of the active genes (RPKM >0.5) as gold standard positives. We identified ERs that have at least 5% overlap with the promoter region (2 kb region around the annotated transcription start site). For this comparison, we sorted the top 20,000 ERs with respect to the score reported by each method then computed the overlap of the ERs with active promoters. Starting from the top ERs, we plotted fraction of active promoters that are identified correctly versus fraction of ERs that overlap with active promoters. These are shown in Figure 3c,d, respectively, for the K562 and GM12878 cell lines. MUSIC performs favorably compared with the other methods, followed by DFilter and SICER. We also compared these methods using the TF CTCF via the enrichment of the known CTCF binding motifs. In this comparison, MUSIC is among the best performing methods (Table S2 in Additional file 1; 'Parameter selection for a new ChIP-Seq dataset' section in Materials and methods).

### Analysis of the RNA polymerase II and gene expression levels

Next, we concentrated on the Pol2 binding data from the ENCODE project. Pol2 shows distinct patterns of binding depending on the state of polymerase, that is, genes with broadly bound polymerase are being actively transcribed (elongating Pol2) and show higher levels of expression compared with genes that are bound in a punctate fashion by Pol2 (stalled Pol2) [34,35]. This makes the Pol2 data suitable for multiscale analysis using MUSIC.

**Figure 4:**
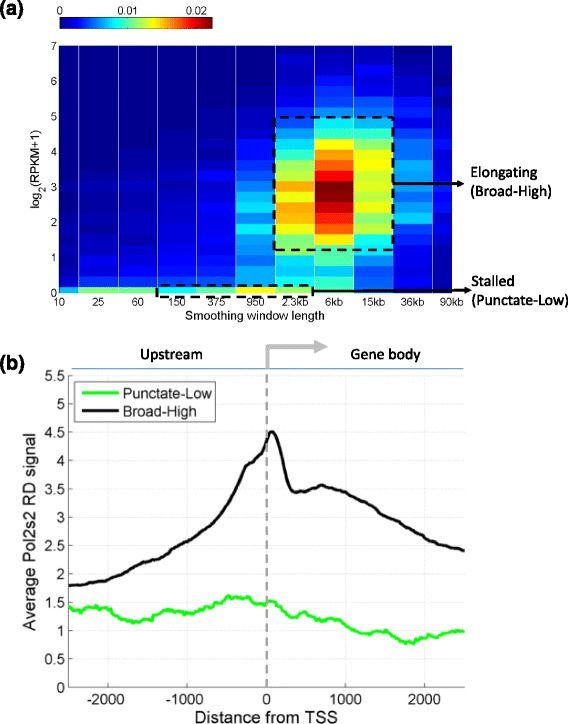
**Distribution of pileup scale versus expression. (a)** Two-dimensional normalized histogram of pileup scale versus log gene expression levels for protein coding genes. The first component, stalled Pol2 binding, is indicated on the graph with 'Stalled'. The second component is indicated on the graph with 'Elongating'. **(b)** Aggregation of Pol2s2 signal around promoters of the genes that are bound by stalled polymerase ('Punctate-Low') and around the promoters of the genes bound with elongating polymerase ('Broad-High').

To evaluate the relation between the expression and the length scale of binding, we processed Pol2 ChIP-Seq data for the K562 cell line from the ENCODE project using MUSIC and computed the SSER pileup scale using parameters *l*_*start*_ = 10 bps, *l*_*end*_ = 2.5 Mbps, and σ = 1.5. Then, for each protein coding gene, we assigned the broadness of Pol2 binding as the maximum of the SSER pileup scale within the gene body. We then quantified the gene expression levels in RPKM using the RNA-Seq datasets from the ENCODE project. Finally, we plotted the two-dimensional histogram of binding scale and gene expression level for each gene (Figure 4a). In the plot, two components are revealed. One component is at low log expression levels (<0.1) and has a maximum frequency at a scale length of 950 bp. This component corresponds to stalled Pol2, which has a punctate enrichment profile and produces very little or no transcripts. The second component is observed at log RPKM <0.5 with a peak of scale level at around 6 kilobases. With the elongating Pol2 and high expression levels, this component is associated with actively transcribed genes.

To study these components further, we focused on the two components of polymerase binding and gene expression levels. For the genes with stalled polymerase, we selected genes with a pileup scale between 150 bp and 2.3 kbp and low expression (log(RPKM) <0.1). For the genes with elongating polymerase, we selected genes with a pileup scale >950 bp with high expression (log(RPKM) >0.1). We performed aggregation of the ChIP-Seq RD signal for the elongating form of polymerase, Pol2s2, from the ENCODE project, around the promoters of genes in both sets. The motivation is that signal for Pol2s2 marks the location of elongating polymerase, which should associate with the promoters that we marked as elongating and not with the promoters that are bound by the stalled polymerase. Figure 4b shows the aggregation plots. As expected, for the punctate bound and low expression genes, the aggregation plot shows very little Pol2s2 binding. In contrast, the high expression and broad bound promoters show substantially higher Pol2s2 binding that extends into the gene body.

## Conclusions

We present a novel method, MUSIC, for the identification of ERs in ChIP-Seq experiments. MUSIC utilizes multiscale decomposition of the ChIP-Seq signal profile in conjunction with a novel mappability correction for mediating the effects of the data. Mappability is an important aspect of ER identification from next-generation sequencing data, especially for identifying broad domains of enrichment since RD profiles are highly correlated with the mappability map. We show that MUSIC outperforms other methods in terms of accuracy of H3K36me3 ERs in comparison with expressed transcripts identified from the expression data from the ENCODE project. An important advantage of MUSIC is that users can specify the scales that they would like to concentrate on, which is done using the begin and end scale parameters for the multiscale filtering. With the diverse enrichment characteristics of the targets for ChIP-Seq experiments, we believe this customizability will prove very useful for processing datasets generated using ChIP-Seq experiments for which broad binding profiles are observed.

Compared with kernel-based linear filters (which are also used in wavelet-based multiscale decompositions), multiscale decomposition using median filtering has two advantages [36]. First, at low noise levels, median smoothing preserves the edges - that is, the sharpness of the increase and decrease of the RD signal at the ends of ERs - in the signal better than linear filters. Second, median smoothing is more tolerant to the burst or impulse noise compared with linear filters. This is important for ER identification since the systematic noise added by multi-mappability can be viewed as an impulse noise [37,38]. In addition to the advantages of median filtering, the preprocessing step of mappability correction also mediates adverse effects of non-uniform mappability. For example, a recent study [39] uses linear Gaussian filtering-based multiscale decomposition to compute multiscale representations of genomic signals. The non-uniform mappability of the genome should be expected to affect the representation since mappability is utilized in a post-processing step after representation is computed, unlike MUSIC, where the mappability correction is performed before decomposition is computed.

We also processed Pol2 data using MUSIC. Pol2 is suitable for multiscale analysis because, unlike other DNA binding proteins, it shows a wide spectrum of ER lengths. Furthermore, the broadness of binding of Pol2 is indicative of its state, that is, stalled or elongating. We showed that there is a significant distinction between the expression levels of genes that are bound broadly by Pol2 compared with genes that are bound in a punctate fashion.

## Materials and methods

We describe the signal processing methodology underlying MUSIC in more detail.

### Input normalization

It is necessary to normalize the control signal profile with respect to the ChIP-Seq profile because the RDs can be different. For each chromosome, MUSIC first divides the chromosome into 10,000 bp bins then computes the total ChIP-Seq and control signal in each window. Finally, it estimates the normalization factors as the slope of the minimum squared error estimate of the slope:

$$ \rho =\underset{\rho \hbox{'}}{\mathrm{argmin}}\left\{{\displaystyle \sum_i{\left({w}_i-\rho \hbox{'}\cdot {c}_i\right)}^2}\right\} $$

where *w*_*i*_ and *c*_*i*_ represent the total signal in the *i*^*th*^ bin for ChIP and control samples, respectively. The normalization procedure aims to match the background signal level in the ChIP sample to the control sample.

### Mappability correction filter

Given the read depth signal at each nucleotide position, MUSIC corrects for the loss of signal caused by low mappability using the following filtering:

$$ {\tilde{x}}_i= \max \left[{x}_i,\ \mathrm{median}\left({\left\{{x}_a\right\}}_{a\in \left[i-{l}_c/2,\ i+{l}_c/2\right]}\ \Big|\ {m}_a<{\overline{m}}_{\mathrm{exonic}}\right)\right] $$

where *x*_*i*_ is the signal value at nucleotide position *i*, median({*x*_*i*_}) is the median of the set {*x*_*i*_}, *m*_*a*_ is the value of the multi-mappability profile at position *a*, and *l*_*c*_ is the window length used in mappability aware filtering. Using this filtering, MUSIC infers the signal values for positions with low mappability using the median of the values at nearby positions with a multi-mappability signal lower than ${\overline{m}}_{\mathrm{exonic}}$, which is 1.2. We selected this value since it is the smallest multi-mappability signal profile value, that is, the most mappable, over exons and promoters as shown in Figure S1 in Additional file 1. We set the window length *l*_*c*_ to 2,000 bp empirically. This window length depends on the distribution of length of the non-mappable region lengths. Different *l*_*c*_ values did not seem to have a significant effect on the results for the human genome.

This filtering procedure is inspired by the dilation operation in image processing, which is a morphological filter and has been used, in combination with other filters, for image enhancement. In our experiments, we also observed that the operation defined above tends to enhance the significant ERs.

### Multiscale decomposition by median filtering

MUSIC utilizes median filtering-based multiscale decomposition. We selected to use median filtering since it has many applications in signal processing for performing signal smoothing with edge preserving. Given a window length, that is, the scale, median filtering can be formulated as:

$$ {x}_i^s=\mathrm{median}\left({\left\{{\tilde{x}}_a\right\}}_{a\in \left[i-\frac{l_s}{2},i+\frac{l_s}{2}\right]}\right),{l}_s\in \left({l}_{start,}\left\lfloor {l}_{start}\times \sigma \right\rfloor, \cdots {l}_{end}\right) $$

where ${x}_i^s$ is the *i*^*th*^ value of the decomposition at scale level *s* for which the smoothing window length is *l*_*s*_, and $\tilde{x}$ is the mappability corrected signal profile. The window length *l*_*s*_ is chosen from a geometric series with the factor *σ* to ensure that the larger scales do not dominate the identified SSERs [21].

The multiscale decomposition enables automatic identification of blobs in the signal profiles at different scales with very small computational requirement. MUSIC uses a fast and efficient method to implement the median filtering by storing the histogram of the signal values in the current window and processing only the new and obsolete signal values that enter and leave the current window to update the histogram when moved to the next window.

### Identification of candidate scale-specific enriched regions

After the multiscale decomposition, MUSIC identifies all local minima in the decomposition. MUSIC utilizes regions between minima points as the regions of enrichment. For this, MUSIC computes the derivative of the signal at each point as the difference between consecutive values:

$$ x{\hbox{'}}_i^s=\left({x}_i^s-{x}_{i-1}^s\right) $$

where $x{\hbox{'}}_i^s$ is the derivative of the smoothed signal ${x}_i^s$. MUSIC assigns the local extrema at the points where the derivative changes sign:

$$ \begin{array}{c}\hfill {I}_{min}=\left\{i\kern0.5em \left|\kern0.5em x{\mathit{\hbox{'}}}_i^s<\kern0.5em 0,\kern0.5em x{\mathit{\hbox{'}}}_{i-1}^s\kern0.5em >\kern0.5em 0\right.\right\}\hfill \\ {}\hfill {I}_{max}=\left\{i\kern0.5em \left|\kern0.5em x{\mathit{\hbox{'}}}_i^s>\kern0.5em 0,\kern0.5em x{\mathit{\hbox{'}}}_{i-1}^s\kern0.5em <\kern0.5em 0\right.\right\}\hfill \end{array} $$

where *I*_*min*_ and *I*_*max*_ are the sets of positions of minima and maxima of ${x}_i^s$, respectively. The scale-specific candidate ERs of ${x}_i^s$ are identified as the regions between the consecutive minima.

### Comparison of smoothed signal in candidate enriched regions

For the candidate ERs in each smoothing scale, MUSIC uses the value of smoothed signal levels and unsmoothed signal levels for assessing the quality of the ERs. A scale-specific candidate ER is filtered if the ratio of the maximum of the smoothed signal to the maximum of the unsmoothed signal within the candidate region is higher than the smoothing statistic threshold, *γ*. In other words, MUSIC removes the candidate ER [*i*, *j*] at scale *s* if:

$$ \frac{ \max \left({\left\{{x}_a^s\right\}}_{a\in \left[i,j\right]}\right)}{ \max \left({\left\{{x}_a\right\}}_{a\in \left[i,j\right]}\right)}<\gamma . $$

The comparison between the ratio on the left and *γ* offers a simple and efficient check to evaluate whether the signal within the candidate region identified at the scale level *s* is severely smoothed. This way, MUSIC efficiently detects and avoids over-merging of consecutive regions that have high signal enrichment and are close to each other. We analyzed the significance of SSERs versus *γ* and determined that a default value of 4 enables a conservative list of SSERs (see the 'Selection of *σ*' section in Materials and methods).

### Candidate enriched region end trimming using a Poisson distribution model

MUSIC trims the ends of the candidate ERs using a Poisson null model for the signal distribution. For this, MUSIC divides the genome into 1 Mbp windows and for each 1 Mbp window estimates the mean of all the values. Using this as the mean parameter *μ* of the Poisson distribution, MUSIC selects a threshold that satisfies a 5% false positive rate:

$$ \tau =\underset{t}{\mathrm{argmin}}\left\{{F}_{X_{\mu }}(t)>0.95\right\},\ {X}_{\mu} \sim Poisson\left(\mu \right) $$

where ${F}_{X_{\mu }}$ represents the cumulative distribution function of *X*_*μ*_, which is distributed as Poisson with mean *μ*. For a region with start and end at positions *i* and *j*, respectively, the trimmed end coordinates are given as:

$$ \begin{array}{l}{i}^{\prime } = \underset{a}{\mathrm{argmin}}\left({x}_a > \tau \right),\ a\ \in \left[i,j\right]\hfill \\ {}{j}^{\prime } = \underset{a}{\mathrm{argmax}}\left({x}_a > \tau \right),\ a\ \in \left[i,j\right]\hfill \end{array} $$

where *i*′ and *j*′ are the trimmed start and end coordinates, respectively. The regions for which the signal level does not pass the threshold are removed from the candidate ER list.

### Candidate enriched region end trimming via *P*-value minimization

MUSIC fine-tunes the ends of the merged ERs using a *P*-value minimization procedure. This maximizes the compactness of the merged regions. The end-refined merged regions are the candidate regions of enrichment before *P*-value computation. The end trimming can be formulated as:

$$ \begin{array}{l}{i}^{\prime } = \underset{a}{\mathrm{argmin}}\left(p\left(a,\ j\ \Big|\ {l}_{p_{val}}=\left(\mathrm{j}-\mathrm{a}+1\right)\right)\right),\ a\ \in \left[i,j\right]\hfill \\ {}{j}^{\prime } = \underset{a}{\mathrm{argmin}}\left(p\left({i}^{\prime },\ a\ \Big|\ {l}_{p_{val}}=\left(\mathrm{a}-\mathrm{i}\hbox{'}+1\right)\right)\right),\ a\ \in \left[i\hbox{'},j\right]\hfill \end{array} $$

where $p\left(a,b\ \Big|\ {l}_{p_{val}}\right)$ represents the *P*-value for the region starting at *a* and ending at *b* with the length of *P*-value window given by ${l}_{p_{val}}$ (Refer to *P*-value computation).

### Per strand concordance test

For each ER, MUSIC computes the total signal on positive and negative strands and filters out the ERs for which there is high discordance between the signals:

$$ \min \left(\frac{{\displaystyle \sum_i{x}_i^{+}}}{{\displaystyle \sum_i{x}_i^{-}}},\frac{{\displaystyle \sum_i{x}_i^{-}}}{{\displaystyle \sum_i{x}_i^{+}}}\right)<0.5 $$

where ${\displaystyle \sum_i}{x}_i^{+}$ and ${\displaystyle \sum_i}{x}_i^{-}$ is the total signal on the positive and negative strand within the start and end coordinates of the ER, respectively.

### *P*-value computation and false discovery rate estimation

We use one-tailed binomial test to compute the *P*-values for each candidate ER. We first count the number of reads in the chip sample (*n*_*chip*_) and control sample (*n*_*control*_) that overlap with the region, then compute one tailed *P*-value as:

$$ p={\displaystyle \sum_{r=n{\hbox{'}}_{chip}+1}^{n{\hbox{'}}_{chip}+n{\hbox{'}}_{conrol}}}\left(\begin{array}{c}\hfill {n}_{chip}^{\hbox{'}}+{n}_{control}^{\hbox{'}}\hfill \\ {}\hfill r\hfill \end{array}\right){0.5}^{\left({n}_{chip}^{\hbox{'}}+{n}_{control}^{\hbox{'}}\right)} $$

where ${n}_{chip}^{\hbox{'}}$ and ${n}_{control}^{\hbox{'}}$ are the normalized read counts for the region:

$$ {n}_{chip}^{\hbox{'}}=\frac{n_{chip}}{l_{chip}}\times {l}_{p_{val}} $$

$$ {n}_{control}^{\hbox{'}}=\frac{n_{control}}{l_{control}}\times {l}_{p_{val}} $$

where ${l}_{p_{val}}$ is the length of the *P*-value computation window and *p* refers to the *P*-value for the ER. It should be noted that the larger values of ${l}_{p_{val}}$ increase the significance of all the regions and the false positive rate (see the 'Parameter selection for benchmarking' section in Materials and methods). We perform multiple hypothesis correction by false discovery rate (FDR) estimation (q-values) using the Benjamini-Hochberg procedure [25]:

$$ {q}_i={p}_i\times \frac{N_{ERs}}{i} $$

where *N*_*ERs*_ is the total number of ERs and *i* is the rank of the ER in the ER list sorted with respect to increasing *P*-value. By default, MUSIC uses a default q-value cutoff of 0.05. The filtered ERs are reported in BED format with their q-values in the score field.

### Summit and trough identification

For DNA-binding protein ChIP-Seq data, for example, TFs, MUSIC reports the location of the highest signal level within the ER as the summit of the signal, which can be used as the binding position. An important consideration in ER identification is the identification of valleys (or troughs) in the signal. For example, the troughs in H3K4me3 and H3K27ac ERs may correspond to the nucleosome-free regions in promoters and enhancers, respectively, where the TFs can interact with DNA and regulate transcription. Therefore, identification of the troughs (in addition to the summits) is an important piece of additional information for each ER. Our analysis, however, shows that many of the troughs in ChIP-Seq signals are caused by a decrease in the mappability of the genome (Figure S6 in Additional file 1). MUSIC reports one trough position in each peak by determining the smallest position within the top two tallest peaks such that the average multi-mappability around the trough is smaller than the exonic multi-mappability (*m*_*e*_). No troughs are reported if there is only one summit in the ER.

### Multi-mappability signal generation

MUSIC can generate multi-mappability signal profiles. For this, MUSIC utilizes an existing read mapping tool. Currently MUSIC uses bowtie2 [40], a very popular short read mapping algorithm, by default. MUSIC first fragments all the chromosomes to the read length of interest, maps all the fragments to the genome using bowtie2 with two mismatches and reporting of a maximum of the top five multimapping positions per fragment. Then MUSIC uses the mapped reads to build the multi-mappability RD signal profile. The regions with high signal correspond to regions with low mappability. We generated multi-mappability profiles for the hg19 genome assembly for read lengths of 36, 50, 76, and 100 bp, which are available for download with MUSIC.

### Parameter selection for benchmarking

Several parameters are associated with MUSIC. We discuss the general selection procedure for these, which can be used as guidelines when running MUSIC.

#### Selection of *l*_*begin*_ and *l*_*end*_

For selecting *l*_*begin*_ and *l*_*end*_, we utilize a basic property of median filtering (Figure S3 in Additional file 1). In order to detect an enrichment of length *l* it is necessary to ensure:

$$ {l}_{begin}<2\times l $$

Similarly, in order to distinguish between two ERs that are *l* base pairs away from each other, it is necessary to ensure:

$$ {l}_{end}<2\times l $$

Thus, *l*_*begin*_ should be small enough to ensure detection of the smallest enrichments that we expect to observe and *l*_*end*_ should be set to a value to detect each individual enrichment separately without over-merging (Figure S3b,c in Additional file 1). As we assume that the basic enriched units are the gene bodies, we choose *l*_*begin*_ using the length distribution of gene bodies (Figure S3e in Additional file 1). As most of the genes are longer than 512 bp (log value of 9), we set *l*_*begin*_ to 1,000 bp. For choosing *l*_*end*_, we computed the cumulative distribution of gene-gene distances (Figure S3d in Additional file 1). Evaluating this plot, we observe that a 10% cutoff occurs at around a log distance of 12.5. As a suitable compromise with the gene length distribution (the median is at a log value of 15), we set *l*_*end*_ to 2 × 2^13^ ≈ 16,000 bp. For punctate marks (like H3K4me3 and H3K27ac), MUSIC is set to run at a smaller scale spectrum than for broader marks using *l*_*begin*_ = 100, *l*_*end*_ = 2,000. This way MUSIC aims to identify small ERs and to identify enrichments at the expected length range of several kilobases. For TFs, where the binding events occur at almost single base pair resolution, MUSIC is set to run at very small scales with *l*_*begin*_ = 100, *l*_*end*_ = 200. It is worth noting that the multiscale decomposition offers the most benefit for the identification of ERs that have a large spectrum of length scales, such as HMs.

#### Selection of ${l}_{p_{val}}$

${l}_{p_{val}}$ tunes the *P*-values of the SSERs and the final set of ERs. This is especially important for broad histone marks (like H3K36me3) because the ERs are observed at a large spectrum of lengths (Figure 2). Generally, increasing ${l}_{p_{val}}$ increases the power of identification (see the '*P*-value computation' section in Materials and methods) but also increases the FDR. In addition, depending on the sequencing depth, ${l}_{p_{val}}$ can be used to avoid saturation of the identified ERs [29]. To select ${l}_{p_{val}}$, we assessed the *P*-values computed using different ${l}_{p_{val}}$ values and fold change (the number of chip sample reads divided by number of normalized control reads). Fold change is generally independent of the sequencing depth and represents an unbiased estimate of enrichment. For different ${l}_{p_{val}}$ values, we divided chromosome 1 into bins of ${l}_{p_{val}}$ base pairs and computed the *P*-value and the fold change in each bin. Figure S4 in Additional file 1 shows the scatter plot of *P*-value versus fold change for different values of ${l}_{p_{val}}$. It can be observed that as ${l}_{p_{val}}$ increases, the *P*-values corresponding to the same fold change decrease. Our basic idea is to choose ${l}_{p_{val}}$ such that the windows that show significant enrichment with respect to fold change (above 2) are also significant with respect to *P*-value (log *P*-value smaller than -3) and that the windows that do not show significant fold change (below 1.5) do not have significant *P*-values. Using these criteria, we set ${l}_{p_{val}}$ to 1,750 bp.

The punctate histone marks (like H3K4me3) and TFs (like CTCF) have much more punctate ERs than broad histone marks. In addition, the ERs are observed at a much smaller spectrum of length scales, especially for the TFs (Figure 2). Therefore, the procedure for selection of ${l}_{p_{val}}$ that we used for broad marks with a large scale spectrum is not very suitable for these marks. Motivated by this, for CTCF, we set ${l}_{p_{val}}$ to 200 bp. H3K4me3 ERs, which mark the promoters, extend several kilobases over the promoters of genes. For H3K4me3, we set ${l}_{p_{val}}$ to 1,500 bp.

#### Selection of *γ*

*γ* is the threshold on the ratio of the maximum of the smoothed signal and the unsmoothed signal on an SSER. This parameter enables MUSIC to avoid over-merging segments by comparing the signal level in the smoothed signal and the original signal. To visualize the effect of changing *γ* on the identified SSERs, we computed the SSERs for the H3K36me3, H3K4me3 and H3K4me1 marks for the K562 cell line. We then computed the smoothing ratio (as defined in the 'Comparison of smoothed signal in candidate enriched regions' section in Materials and methods) for each SSER. Then we plotted the cumulative distribution of all the SSERs with respect to the smoothing statistic (Figure S5 in Additional file 1). For H3K4me3 and H3K4me1, it can be seen that the distribution is more skewed toward smaller *γ* than for H3K36me3, which is expected since these marks have much narrower ERs than H3K36me3. To be as inclusive as possible, we choose *γ* = 4 (around 98% of the SSERs for H3K4me3 and H3K4me1, and 90% of the SSERs for H3K36me3 pass the smoothing statistic test) as a suitable parameter to balance the tradeoff between being inclusive in the identified SSERs and over-merging the ERs.

#### Selection of *σ*

The final parameter to set is *σ*, which is the multiplicative factor between the consecutive scales. Higher values of *σ* decreases the runtime of MUSIC but important information can be lost since sampling of the scale space is sparsified. For example, SSERs that can be identified at a mid-scale can be lost. We evaluated several different values for *σ* and observed that, for *σ* >2, MUSIC uses a very sparse set of scales that miss many ERs. As a suitable compromise, we chose to use *σ* = 1.5. It should be noted that it may be useful to use smaller values for *σ* when more punctate ERs are being analyzed. For example, for a more detailed analysis of the scale space, *σ* = 1.1 can be used to perform the scale spectrum analysis in Figure 2.

### Parameters used for peak calling methods in benchmarking

The most recent versions of the tools can be downloaded from their respective websites and the documentation for each should be followed for running the tool in the correct mode.

#### BCP

For histone marks (H3K36me3, H3K27me3, and H3K4me3), we used the BCP_HM tool with command line options: -f 200 -w 200 -p 0.05. For the CTCF dataset, we used the BCP_TF tool with command line options: -e 10 -p 0.00000001.

#### PeakRanger

For histone marks, we used the ‘ccat’ option for broad peak calling. For CTCF peaks, we used the ‘ranger’ option.

#### ZINBA

For broad histone marks (H3K36me3, H3K27me3), we used the unrefined ERs from ZINBA with the ‘broad’ flag on as explained in the documentation. For H3K4me3 and CTCF peaks, we used the refined peaks with the ‘broad’ flag turned off.

#### F-Seq

For histone marks and CTCF, F-Seq was run in the default mode.

#### SICER

For histone marks, SICER was run with the command options: hg19, w = 200, fragment_size = 150, 0.74, g = 600, FDR = 0.01. For CTCF, SICER was run with smaller gap size of g = 200.

#### SPP

For broad marks, SPP was run in broad mode using get.broad.enrichment.clusters(…). For CTCF, the peak calling mode was run using find.binding.positions(…).

#### DFilter

For H3K36me3 and H3K27me3, DFilter was run with the command line options ‘-nonzero -bs = 100 -ks = 30 -std = 2’ and peaks that had a score <2 were removed. For H3K4me3, DFilter was run using ‘-bs = 100 -ks = 100 -dir -std = 2’ and peaks that had a score <6 were removed. For CTCF, we ran DFilter with ‘-bs = 50 -ks = 30 -refine -nonzero -std = 2’.

#### MACS

For histone marks, MACS was run with options ‘--broad -g hs’. For CTCF, MACS was run with ‘-g hs -q 0.01’.

### Parameter selection for a new ChIP-Seq dataset

When a new ChIP-Seq experiment is performed for a factor or HM that is not included in this study, it is necessary to estimate the parameters *l*_*begin*_, *l*_*end*_, ${l}_{p_{val}}$ , and γ. Computation of the scale spectrum is of central importance for characterizing the new dataset and selecting parameters for analysis with MUSIC. For this, a large scale spectrum is scanned (for example, 100 to 1,000,000 bp) using a small value for *σ* (for example, 1.1) and an initial value for ${l}_{p_{val}}$. The initial selection of ${l}_{p_{val}}$ should follow the procedure outlined above ('[Sec Sec25]' section) and set to a stringent value to decrease the false positive rate for SSERs. The scale spectrum can be plotted as in Figure S7 in Additional file 1. After the scale spectrum is generated, the parameter selection follows.

For selection of *l*_*begin*_ and *l*_*end*_, the scale spectrum is evaluated and if the dataset can be classified as either punctate or broad, the values for *l*_*begin*_ and *l*_*end*_ that are set as described above ('Selection of *l*_*begin*_ and *l*_*end*_' section) can be utilized. When the spectrum is not similar to any of the HMs studied here, *l*_*begin*_ can be set to the smallest length scale at which there is a significant fraction of ERs in Figure S7 in Additional file 1. In general, however, the signal to noise ratio is smaller for ERs identified at smaller scales than for ERs identified at larger scales. It is therefore useful to set *l*_*begin*_ to a high enough value to decrease the fraction of false positive ERs. Knowledge of the smallest expected ER length may be useful for this. For setting *l*_*end*_, it is necessary to estimate the distance between neighboring ERs to ensure that there is no over-merging in the decomposition. To accomplish this, MUSIC assesses the significantly enriched regions in the scale spectrum and estimates the mean ER to ER distance. This distance can be used as described above ('Selection of *l*_*begin*_ and *l*_*end*_' section) to set *l*_*end*_. It is also important to note that the scale length at which the scale spectrum (Figure S7 in Additional file 1) has a global maximum should be definitely larger than *l*_*end*_. Thus, the following formula is useful for setting *l*_*end*_:

$$ {l}_{end}= \min \left(2\times {\overline{l}}_{ER-ER},\ {l}_{\mathrm{spectrum}}\right) $$

where ${\overline{l}}_{ER-ER}$ is the estimate of the mean ER to ER distance and *l*_spectrum_ is the scale length at which the scale spectrum has a maxima. Although the above criteria can be used to select the natural scale of any HM, it is also important to note that one can choose to analyze the enrichments at a different scale for studying biological phenomena at different scales. For example, a very large scale analysis (for example, around 100 kbp scale) of H3K36me3 can reveal the large segments of the genome that are active, which may be associated with the positions of the transcription factories [41].

For selection of ${l}_{p_{val}}$ (for identification of SSERs and the final set of ERs), the analysis detailed above ('[Sec Sec25]' section) can be performed. For identification of a suitable maximum length for ${l}_{p_{val}}$, the shape of the scale spectrum is useful (Figure 2). If there is a distinguishable single peak in the spectrum (for example, H3K4me3, H3K4me1 or CTCF), the HM or factor can be thought to be dominated by the length scale corresponding to the peak and ${l}_{p_{val}}$ should be bounded by that value. For datasets with a larger scale spectrum of ER lengths (for example, H3K36me3, H3K27me3, H3K9me3, Pol2b) the maximum for ${l}_{p_{val}}$ should not be much larger than *l*_*begin*_ as the minimum ER length is going to be related implicitly to *l*_*begin*_ (Figure S3 in Additional file 1). In general, increasing ${l}_{p_{val}}$ increases sensitivity and also the false positive rate.

The threshold for the smoothing statistic, *γ*, should not be changed from the value of 4 as it can be seen in Figure S5 in Additional file 1 that *γ* =4 is a fairly inclusive threshold (in order to maximize the power of detection) of the smoothing statistic for both broad and punctate ERs. In case the user chooses to select *γ* for a more stringent specificity at the expense of sensitivity, an analysis similar to the analysis presented above ('Selection of *γ*' section) will be useful to identify the value of *γ* for a given sensitivity at a more stringent *P*-value threshold.

### Accuracy measures

For evaluating the accuracy of H3K36me3 ER calls, we computed sensitivity and positive predictive values:

$$ Sensitivity=\frac{covg\left(P{\displaystyle \cap }G\right)}{covg(G)} $$

$$ PPV=\frac{covg\left(P{\displaystyle \cap }G\right)}{covg(P)} $$

where *covg*(*P*) is the coverage of ERs, *covg*(*G*) is the coverage of expressed gene bodies and *covg*(*P* ∩ *G*) is the coverage of the overlap between expressed gene bodies and ERs. We combined these two accuracy measures to compute the F-measure:

$$ F- measure=\frac{2\times Sensitivity\times PPV}{\left( Sensitivity+PPV\right)} $$

For assessing the reproducibility of the identified ERs from two biological replicates, we use the average overlap fraction between the ERs:

$$ Overlap\  Fraction=\left(\frac{covg\left({P}_1{\displaystyle \cap }{P}_2\right)}{2\times covg\left({P}_1\right)}+\frac{covg\left({P}_1{\displaystyle \cap }{P}_2\right)}{2\times covg\left({P}_2\right)}\right) $$

where *covg*(*P*_1_) and *covg*(*P*_2_) represent the coverage of the ERs identified from replicate 1 and replicate 2, respectively.

For H3K4me3 ER accuracy assessment, we sorted the top 20,000 ERs identified by each method. Then we overlapped the identified ERs with the promoters of active genes (RPKM >0.5), which are defined as the 2,000 bp in the vicinity of the annotated transcription start site. We enforced that the overlap between the promoter region and the peaks was at least 5% of the length of the peak. Then, starting from the top 1,000 ERs, we computed the fraction of active promoters recovered and the fraction of ERs that overlap with active promoters for the top peaks. At each step, we increased the peak number by 1,000.

For CTCF peaks, we sorted the top 2,000 peaks from each method, then computed the fraction of peaks whose summit overlaps within 150 bp of a known CTCF motif.

### Datasets and data processing

The ChIP-Seq datasets for H3K36me3, H3K27me3, H3K4me3, H3K4me1, H3K27ac, and H3K9me3 modifications, Pol2, and CTCF were obtained from ENCODE [5] through the UCSC genome browser. The accession codes for these datasets are GSM733714, GSM733679, GSM733680, GSM733708, GSM733658, GSM733758, GSM733692; GSM733656, GSM733776, GSM733643. The transcript quantifications and RNA-Seq datasets were downloaded from Djebali *et al*. [33] with accession number GSM765405. For the transcript quantifications, we used the average RPKM values for the transcripts from two replicates that satisfied the reproducibility criteria that the irreproducible discovery rate (iIDR) of the gene expression quantification is smaller than 0.1. The transcript and gene annotations were obtained from Harrow *et al*. [42]. The CTCF motifs were downloaded from [43].

## Additional file

Additional file 1
**Supplementary text, Tables S1 and S2, and Figures S1 to S7.**

## Abbreviations

bp: base pair
ER: enriched region
FDR: false discovery rate
HM: histone modification
Pol2: RNA polymerase II
RD: read depth
SSER: scale-specific enriched region
TF: transcription factor
