# Supplementary material for: MUSIC: identification of enriched regions in ChIP-Seq experiments using a mappability-corrected multiscale signal processing framework
Source: Genome Biol. 2014 Oct 8;15(10):474. doi: 10.1186/s13059-014-0474-3 (PMC4234855; doi:10.1186/s13059-014-0474-3)
Supplement: Additional file 1 — Supplementary text, Tables S1 and S2, and Figures S1 to S7. [file 13059_2014_474_MOESM1_ESM.pdf]

# SUPPLEMENTARY MATERIAL

## 1 Multi-Mappability Signal Aggregations

We used the multi-mappability signal profiles generated by MUSIC (See Methods) and aggregated the signal on different regions. Figure S1 shows the aggregation plots of multi-mappability signal over protein coding gene promoters (TSS +/- 2.5 kbs), randomly selected set of introns and exons, and randomly selected regions on the human genome. Promoters, exons, and the regions downstream of TSS into the first exon show significantly higher mappability compared to random regions. In addition, introns show slightly higher mappability compared to random regions and exons show are much more mappable than random regions. Transcription start sites and mid points of exons show almost the same amount of average multi-mappability, 1.2 reads.

## 2 Motivational Example for Multiscale Decomposition

Figure S2 illustrates the 4 level multiscale decomposition of histone modification read depth signal profile. The minima and maxima of the decomposition are indicated on the signal. As the scale level increases, the large scale structures are revealed in the signal. The 3 broadly enriched regions are revealed at the 4$^{th}$ scale as 3 "blobs", which can be identified as the regions between local minima regions.  The smaller scales contain more detailed structure in the signal.

## 3 Statistics on ERs Identified by Different Methods

Table S1 shows the count and coverage for the enriched regions (ERs) for H3K36me3 datasets identified by different methods.

## 4 Fraction of CTCF Peaks with CTCF Motifs

Table S2 shows the fraction of CTCF peaks (for each compared method) for which there is a known CTCF motif within the 150 bp vicinity of the summit reported by the methods.

## 5 Multi-mappability Signal  Aggregation on MACS Specific Troughs

For comparing the regions that are merged by MACS and not merged by MUSIC, i.e., the troughs in the signal specific to MACS, we used the H3K36me3 ERs identified by each method. We first overlapped the ERs that are called in both. Then we subtracted the overlapping MACS ERs from the overlapping MUSIC ERs to generate a set of ERs that are not called by MACS but are called by MUSIC. These remaining regions are used as the troughs specific to MACS. Then, to evaluate the mappability of these MACS specific troughs, we aggregated the multi-mappability signal on these regions. For aggregation, we use the center point of each trough and extended the region 2500 base pair to each side. Finally, we generated set of control regions by translating all the MACS specific troughs 10000 base pairs to right.

Figure S6 shows the aggregation plots on the MACS specific troughs and for the control regions. It can be seen that MACS specific troughs show a significant decrease in the mappability.

# 6 Scale Specific ER Identification and Scale Spectrum Analysis

The scale length spectrum for a new HM (or any other ChIP-Seq data) can be generated in multiple ways. First is via pileup of the SSERs and counting the number of SSERs at each scale as explained in the Section 2.1.2. Here, we explain another method to generate the scale spectrum. Both of the methods can be utilized for identifying a scale length for a new ChIP-Seq data with unknown length scale for the ERs Given a set of SSERs identified from a large scale lengths, we first take the union all the SSERs from a large spectrum of multiscale decompositions, then for each merged region, we intersect the merged regions with the SSERs and assigning a scale to the merged region by finding the largest scale at which an SSER overlaps with the merged region. This way the merged regions are assigned to a scale length. Afterwards, the scale spectrum generated by computing the coverage of the merged regions for each scale level. The scale spectrum for several HMs are shown in Fig S7. It can be observed immediately that each HM has an associated dominating scale length. We observed that this procedure in general gives a cleaner spectrum for identification of dominating scale length compared to Fig 2 to be used in parametrization of scale lengths.

# Table S1

| | K562 ERs | | GM12878 ERs | |
|---|---|---|---|---|
| | Number | Coverage (base pairs) | Number | Coverage (base pairs) |
| MUSIC | 16,768 | 390,296,556 | 13,704 | 469,576,029 |
| BCP | 10,521 | 347,394,000 | 10,190 | 393,830,600 |
| SICER | 40,352 | 314,022,848 | 33,817 | 349,352,583 |
| SPP | 76,215 | 269,242,136 | 75,675 | 278,092,847 |
| PeakRanger | 96,966 | 209,984,550 | 117,836 | 275,866,850 |
| MACS | 62,201 | 125,919,021 | 46,515 | 146,737,896 |
| DFilter | 5,688 | 224,967,800 | 2,520 | 94,333,200 |
| F-Seq | 510,235 | 86,053,106 | 619,748 | 107,425,384 |
| ZINBA | 33,594 | 359,815,781 | 31,562 | 462,042,813 |

**Table S1: The number and coverage of H3K36me3 ERs identified by each method.**

# Table S2

| | Fraction of K562 ER summits within 150 bps of CTCF Motif | Fraction of GM12878 ER summits within 150 bps of CTCF Motif |
|---|---|---|
| MUSIC | .716 | **.725** |
| BCP | .695 | NA |
| SICER | .398 | .440 |
| SPP | .708 | .725 |
| PeakRanger | .430 | .472 |
| MACS | .716 | .721 |
| DFilter | **.726** | .720 |
| F-Seq | .625 | .678 |
| ZINBA | .540 | .529 |

**Table S2: The fraction of top 2000 CTCF peaks that have a known motif within 150 base pairs of the reported summit. The methods are shown on the rows.**

# Figure S1

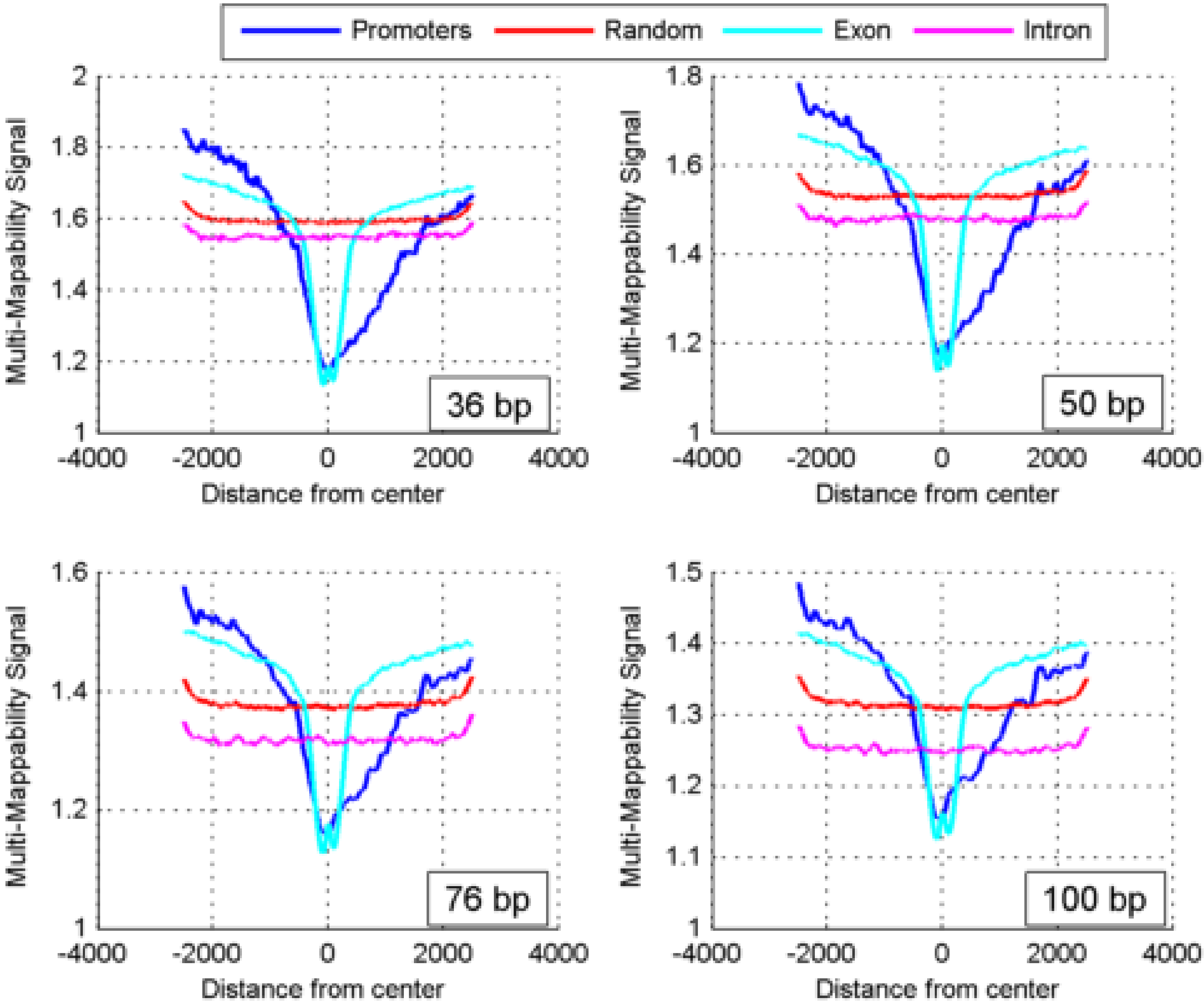


**Figure S1: The aggregation of the multi-mapability signal profiles over promoters, exon, introns, and random regions with at least 1 read that is mapped in any control dataset. 4 different read lengths, as indicated in the textbox in bottom right corner.**

# Figure S2

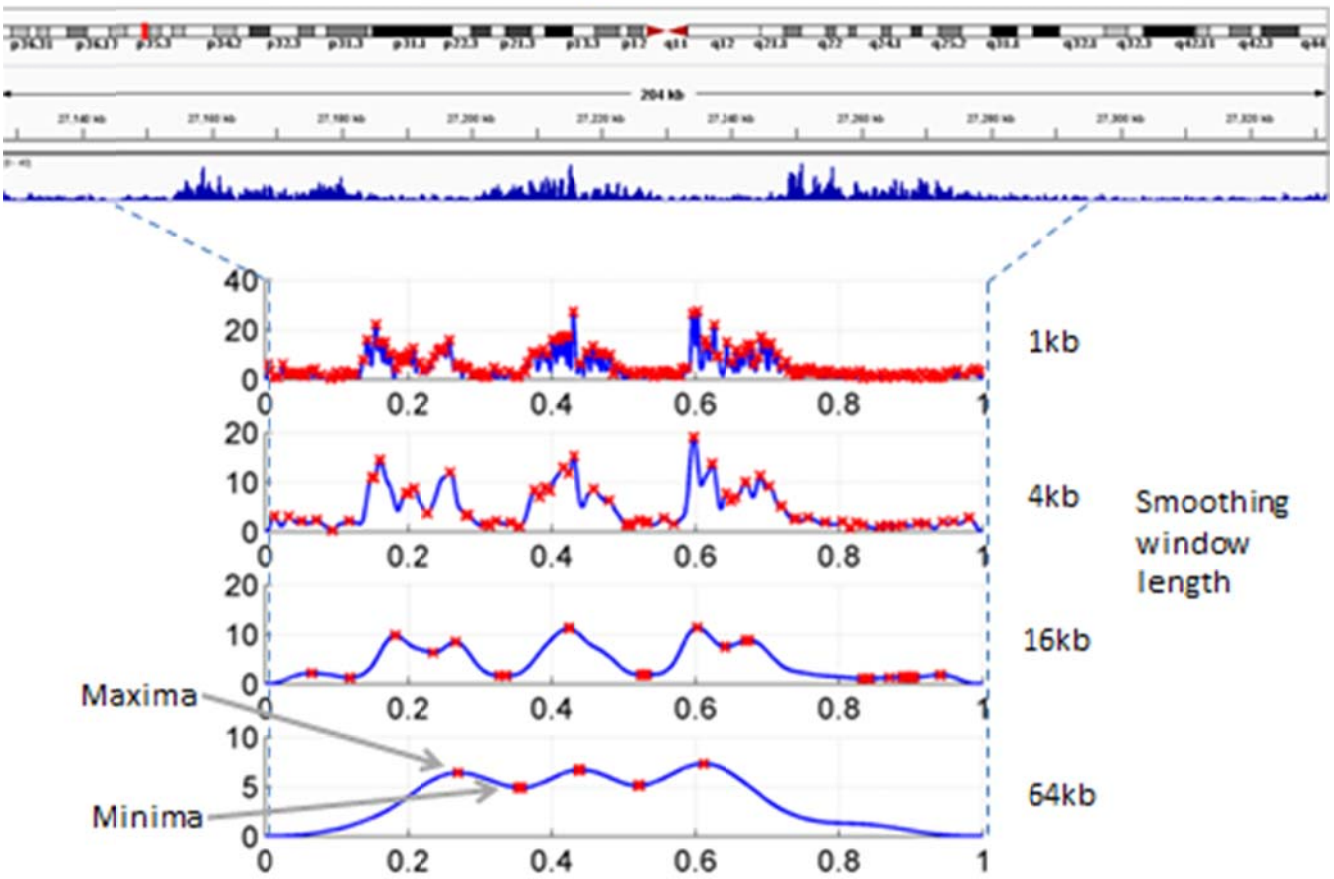


**Figure S2: Illustration of smoothing process. 4 scale decomposition of HM signal using 1 kb, 4kb, 16 kb and 64 kb long smoothing windows. The local extrema in the decompositions are highlighted with red markers on each smoothed signal. The three broad peaks can be identified with the 3 maxima at the decomposition scale with smoothing window length 64kb.**

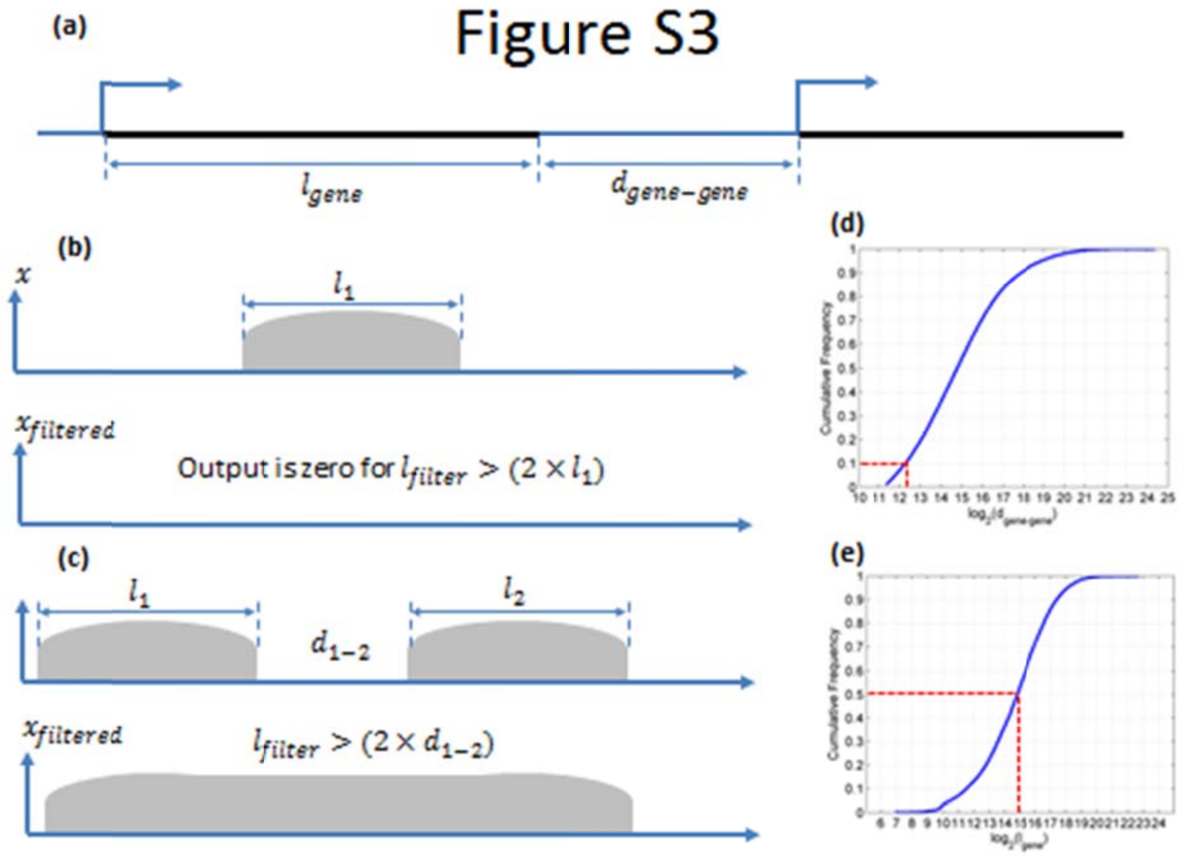


**Figure S3: Illustration of gene-gene distance computation (a). Illustration of failure of detection when the smoothing window length (filter length) is longer than the twice the size of gene body (b). Illustration of overmerging when the smoothing window length is longer than twice the size of gene-gene distance (c). The cumulative distribution plot for intergene distance for genes that are at least 2.5kb apart (d). The cumulative distribution plot for gene lengths from protein coding gene annotations from GENCODE (e).**

# Figure S4

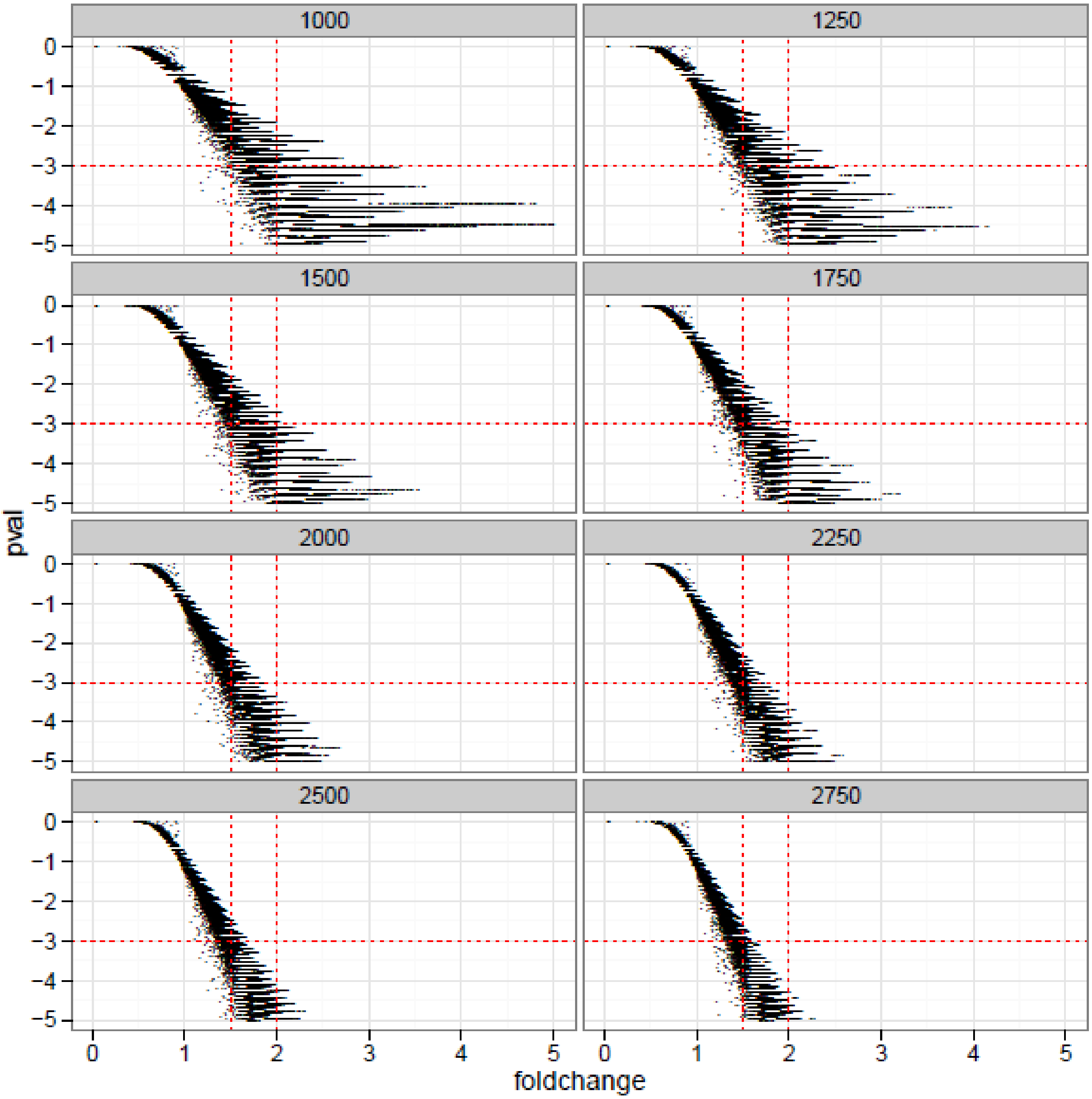


**Figure S4: The plot for p-value versus fold change using the different p-value normalization window lengths. Fold changes 1.5 and 2 are indicated with vertical red dashed lines and p-value of 0.05 (log p-value of -3) is marked with horizontal red dashed line.**

# Figure S5

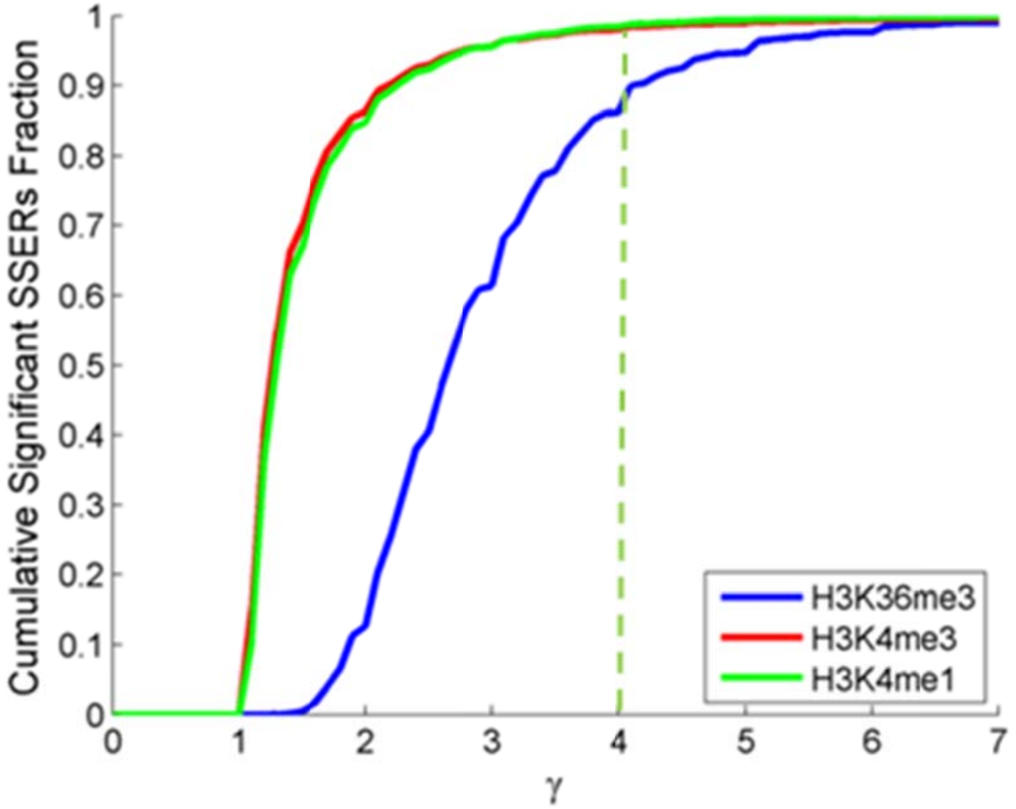


**Figure S5: The cumulative distribution of the significant SSERs with respect to increasing γ. Y-axis shows the cumulative fraction of SSERs whose smoothed versus unsmoothed signal ratio is smaller than γ on x-axis.**

# Figure S6

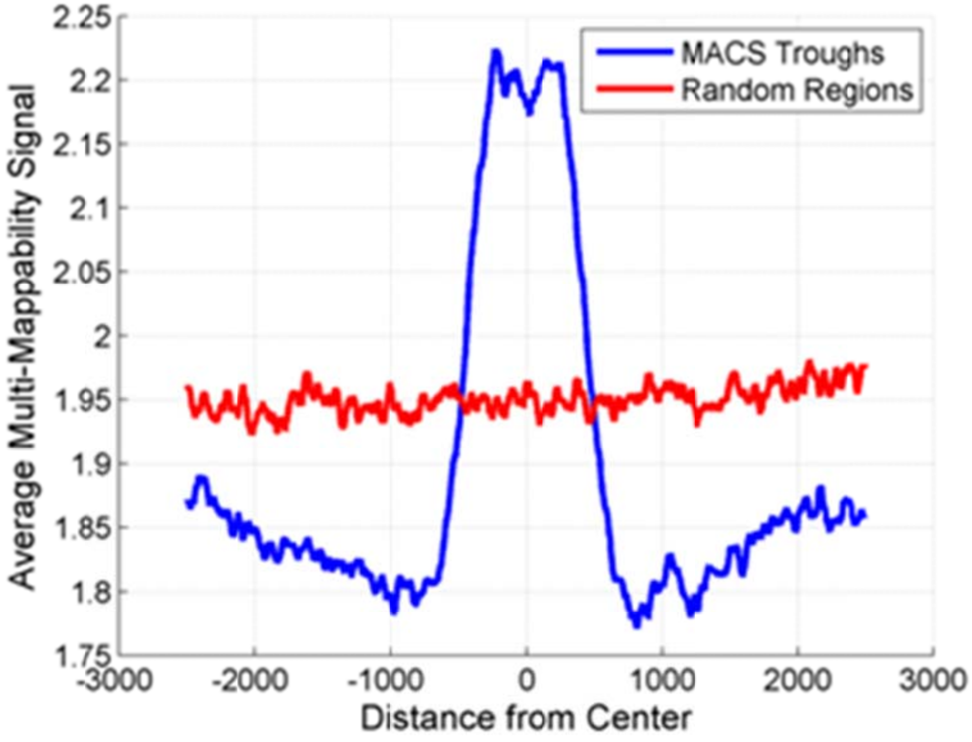


**Figure S6: The aggregation of multi-mappability signal over the MUSIC H3K36me3 ERs that are not identified by MACS (Marked with 'MUSIC specific'). X-axis shows the distance from the mid-point of the region. The random regions are generated by translating the MACS specific regions to 3' end by 10,000 bps.**

# Figure S7

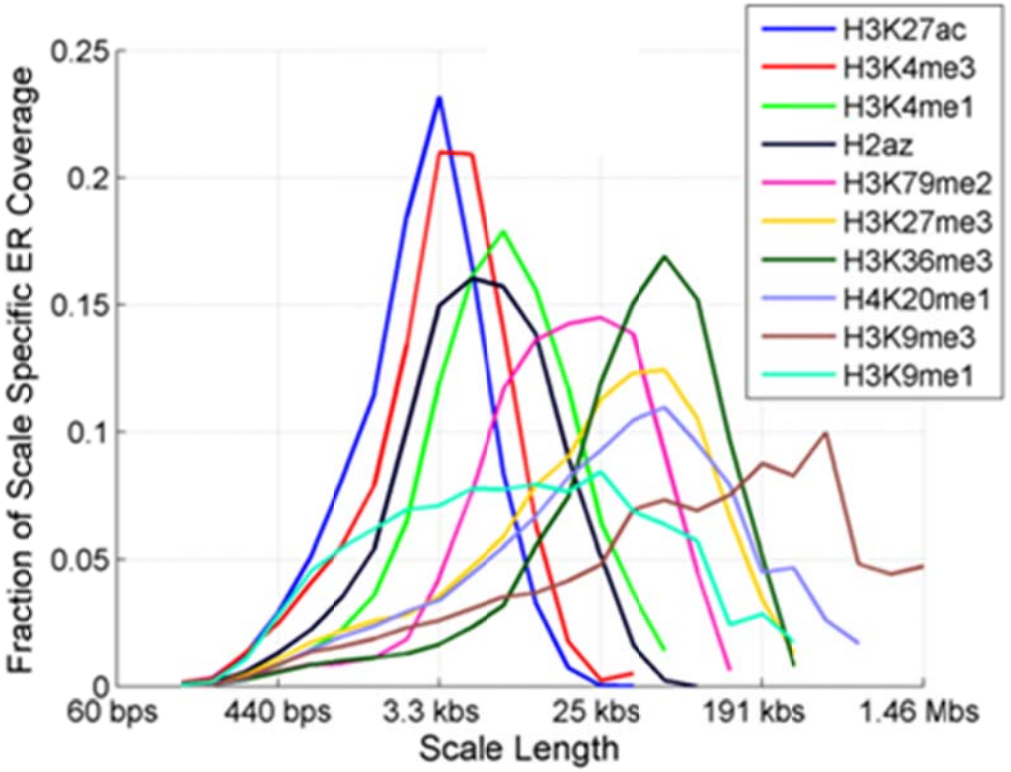


**Figure S7: The scale spectrum for HMs computed using ER specific scale spectrum computation procedure (See Section 6 in Supplementary Materials). X-axis is the scale length, y-axis is the fraction of coverage for the observed scale length. Each HM has a specific scale at which they are enriched, which is utilized in setting the scale length parameters. See Section S6 for more details.**
